# Anteroinferior Psoas Technique for Oblique Lateral Lumbar Interbody Fusion

**DOI:** 10.1111/os.12930

**Published:** 2021-05-05

**Authors:** Hai‐Feng Zhu, Xiang‐Qian Fang, Feng‐Dong Zhao, Jian‐Feng Zhang, Xing Zhao, Zhi‐Jun Hu, Shun‐Wu Fan

**Affiliations:** ^1^ Department of Orthopaedics, Sir Run Run Shaw Hospital School of Medicine, Zhejiang University Hangzhou China; ^2^ Key Laboratory of Musculoskeletal System Degeneration and Regeneration Translational Research of Zhejiang Province Hangzhou China

**Keywords:** Anteroinferior psoas; Direct visualization; Oblique lateral lumbar interbody fusion; Retractor; Retroperitoneal anatomic corridor

## Abstract

Oblique lateral lumbar interbody fusion (OLIF) has been extensively used, with satisfactory outcomes for the treatment of degenerative lumbar disease. This article aims to demonstrate a modified lateral approach, also known as the anteroinferior psoas (AIP) technique for OLIF, which is expected to enhance security by operating under direct vision. The core procedures of our technique are as follows. First, a minimal skin incision is recommended 2 cm backward compared with the normal incision of OLIF, facilitating the oblique placement of the working channel and the orthogonal maneuver for the cage placement. Second, two special custom‐made retractors, as an alternative to the index finger, are used to pull the psoas muscle to the dorsal side and pull the abdominal organs together with extraperitoneal fate to the ventral side under direct visualization, making the exposure of the working channel convenient and safe and avoiding radiation exposure. Third, the anterior border of the psoas is bluntly dissected and retracted backwards, obviously enlarging the retroperitoneal anatomic corridor and then expanding clinical indications of OLIF. The benefits of this technique include that it has a short learning curve, satisfactory clinical outcomes, and low risk of perioperative complications.

## Graphical abstract

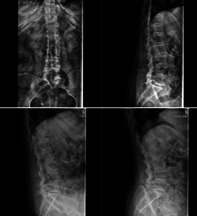

## Introduction

Oblique lateral interbody fusion (OLIF) is being widely used as an alternative to lateral lumbar interbody fusion (LLIF), which is associated with a high risk of access‐related psoas muscle injury and lumbar plexus injury while dissecting the psoas muscle^1^, ^2^. The OLIF procedure, taking advantage of the anatomical space between the aorta and psoas muscle to access to the disc space, can effectively reduce the risk of access‐related neurologic and muscular complications^3^, ^4^. Nevertheless, with the popularization and development of the OLIF technique, more and more surgeons are finding it hard to complete OLIF operations safely, especially beginners. First, as the anatomical space to access the L_2–5_ discs, fluctuating from 15.00 mm to 19.25 mm for Westerners^5^ and 9 mm to 13 mm for Chinese, is a little smaller than the diameter of the Medtronic METRx tube (22 mm) and the width of the PEEK cage (18 mm), it is not an easy task to settle the Medtronic METRx tube safely just using the retroperitoneal anatomic corridor. Second, according to a previous technical report^6^, ^7^, a blunt dissection using an index finger was recommended to expose the retroperitoneal space: applying back‐and‐forth and up‐and‐down movements until the anterior psoas border and intervertebral space was felt. This non‐direct visual exposure may endanger the anterior big vessels, the segmental arteries, the ovarian/testicular veins, the peritoneum and the ureter^8^. Hence, we propose a modified lateral approach, also known as the anteroinferior psoas (AIP) technique for OLIF, which is expected to enhance security by operating under direct vision.

## Technique

### Case study

A 62‐year‐old woman had suffered from recurrent low back pain accompanied by aching pain of the left thigh, which was aggravated during activity and relieved after rest, 3 years prior to her presentation to our hospital. The disease had progressed slowly, but it had become worse suddenly, 3 months prior, without apparent cause. An obvious tenderness and step‐like feeling at L_4–5_ level was found on physical examination by the attending physician. The patient had no knowledge of any underlying disease.

**Fig. 1:**
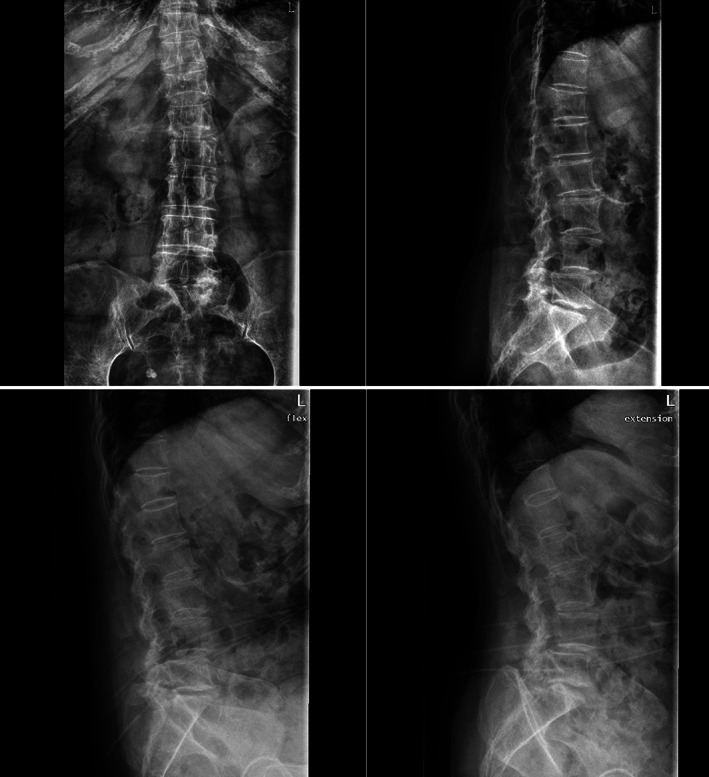
Preoperative static and dynamic anteroposterior radiograph of the lumbar spine. Mild forward slippage of L_4_, which manifested as instability in lumbar dynamic position, scoliosis, and degenerative change with osteoporosis.

**Fig. 2:**
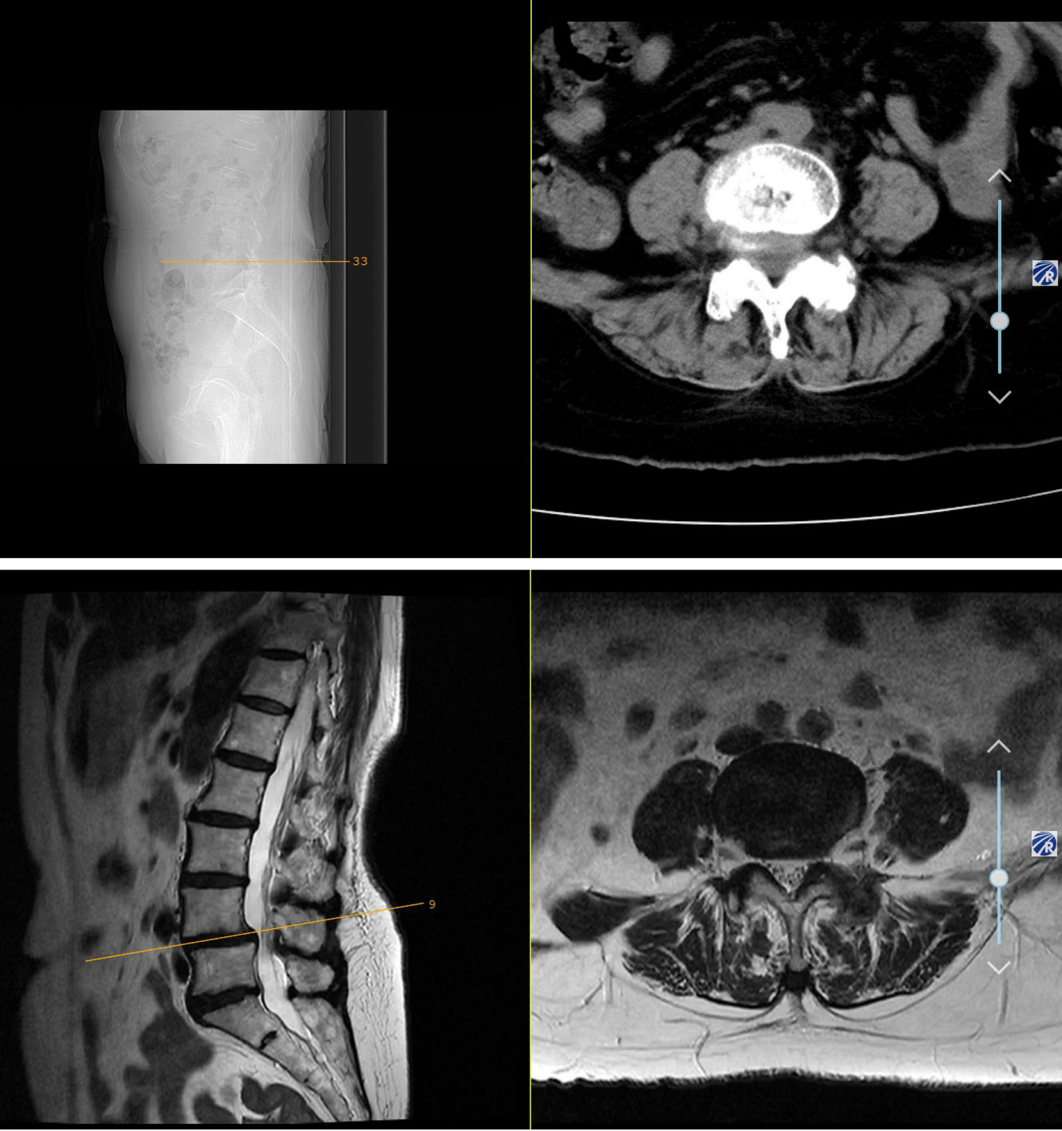
Preoperative lumbar CT scan and MRI. I degree spondyloisthesis of L_4_ and spinal canal stenosis at the L_4–5_ level.

Plain X‐ray images of the lumbar spine revealed mild forward slippage of L_4_, which manifested as instability in lumbar dynamic position, scoliosis, and degenerative change with osteoporosis (Fig. 1). Lumbar CT scan and MRI both showed I degree spondylolisthesis of L_4_ and spinal canal stenosis at L_4–5_ level (Fig. 2).

The diagnosis was lumbar instability accompanied with L_4_ degenerative spondylolisthesis (I degree) and spinal canal stenosis. Strict conservative treatment for more than 3 months was unsuccessful and oblique lateral lumbar interbody fusion was necessary.

### Surgical Technique

The surgical procedure of the AIP technique for OLIF is briefly described in what follows.

#### Position and Incision

After induction of general anesthesia, the patients were positioned in the lateral decubitus position on their right side. Lateral and anteroposterior C‐arm fluoroscopic images were obtained to confirm the disease segment and the central point of the target intervertebral disc (IVD) space. A minimal skin incision is recommended approximately 3–4 cm anterior to the midpoint of the target IVD and 3 cm in length.

#### Exposure of The Target Intervertebral Disc Space with the Anteroinferior Psoas Technique

The obliquus externus abdominis and obliqus internus abdominis were bluntly dissected along the direction of the muscle fiber, and the transverses abdominis was incised. Then the retroperitoneal space was bluntly dissected and the peritoneum was mobilized anteriorly using a special custom‐made retractor to expose the anterior border of the psoas. The IVD was identified by retracting the anterior border of the psoas posteriorly using a periosteum detached under direct visualization, and then the psoas muscle was dissected from the disc surface and retracted posteriorly using another special custom‐made retractor.

#### Establishment of the Working Channel

The guide pin, probe, sequential dilators, and the tube retractor were sequentially placed in the disc space vertically, and the retractor was fixed to the upper bone endplate of the inferior vertebral body with a pin.

#### Discectomy, Endplate Preparation, and Cage Placement

Discectomy and endplate preparation were performed and the opposite annulus fibrosus was knocked through using sequential reamers. The disc space was sequentially distracted by trial until adequate disc space height was obtained, and a peek cage (Clydesdale Spinal System, Medtronic Sofamor Danek, Minneapolis, MN, USA) filled with artificial bone (Wright, Tennessee, USA) was inserted vertically into the intervertebral space.

#### Pedicle Rod Instrumentation Placement

After the anterior procedure, the patient was turned to the prone position and underwent posterior fixation through the inter‐muscular Wiltse procedure with the help of two micro‐laminectomy retractors if necessary.

## Discussion

There is a general consensus that OLIF has several distinct advantages, such as reducing the need to penetrate the psoas and lumbar plexus^2^, avoiding damage to the neural canal, the paraspinal muscle and the posterior ligament complex^9^, larger cage placement to improve fusion rates, and relatively broad indications for treatment. However, a steep learning curve and potentially serious complications, such as abdominal great vascular injury^10^, obviously hinder the uptake of the OLIF technique. Based on our previous study^11^, the AIP technique for OLIF demonstrated a short learning curve, satisfactory clinical outcomes, and low risk of perioperative complications, with surgical procedures completed under direct visualization.

### Highlights and Pitfalls

1. Compared with the normal incision of OLIF, the skin incision is recommended to be 2 cm backward, facilitating the oblique placement of the working channel and the orthogonal maneuver for the cage placement.
2. Two special custom‐made retractors, as an alternative to the index finger, are used to dissect and pull the psoas muscle to the dorsal side and pull the abdominal organs together with the extraperitoneal fate to the ventral side^7^ ^,^ ^11^. Hence, the entire working channel can be directly visualized, which will certainly decrease potential risk to the ureter, sympathetic chain, peritoneum, and vascular structures and reduce the frequency of intraoperative fluoroscopy.
3. The anterior border of the psoas is bluntly dissected and retracted backwards, making the retroperitoneal anatomic corridor obviously enlarged, which will enable some patients with a narrow anatomical corridor to undergo OLIF operations.
4. Although vascular injury is rare, when it does present it can be catastrophic. The following advice could be useful when encountering vascular injury: (i) pressing the two ends of the damaged vessels with two periosteal detachers; (ii) properly dissociating the two ends of the damaged vessels and vascular ligation or bipolar electrocoagulation hemostasis; and (iii) checking again and confirming that there is no active bleeding after removal of the two periosteal detachers.

## Video Image

Additional video images can be found in the online version of this article.
